# The Continuing Evolution of HIV-1 Therapy: Identification and Development of Novel Antiretroviral Agents Targeting Viral and Cellular Targets

**DOI:** 10.1155/2012/401965

**Published:** 2012-07-10

**Authors:** Tracy L. Hartman, Robert W. Buckheit

**Affiliations:** Anti-Infective Research Department, ImQuest BioSciences, Inc., 7340 Executive Way, Suite R, Frederick, MD 21704, USA

## Abstract

During the past three decades, over thirty-five anti-HIV-1 therapies have been developed for use in humans and the progression from monotherapeutic treatment regimens to today's highly active combination antiretroviral therapies has had a dramatic impact on disease progression in HIV-1-infected individuals. In spite of the success of AIDS therapies and the existence of inhibitors of HIV-1 reverse transcriptase, protease, entry and fusion, and integrase, HIV-1 therapies still have a variety of problems which require continued development efforts to improve efficacy and reduce toxicity, while making drugs that can be used throughout both the developed and developing world, in pediatric populations, and in pregnant women. Highly active antiretroviral therapies (HAARTs) have significantly delayed the progression to AIDS, and in the developed world HIV-1-infected individuals might be expected to live normal life spans while on lifelong therapies. However, the difficult treatment regimens, the presence of class-specific drug toxicities, and the emergence of drug-resistant virus isolates highlight the fact that improvements in our therapeutic regimens and the identification of new and novel viral and cellular targets for therapy are still necessary. Antiretroviral therapeutic strategies and targets continue to be explored, and the development of increasingly potent molecules within existing classes of drugs and the development of novel strategies are ongoing.

## 1. Introduction

Since the approval of AZT for the treatment of HIV-1 infection, twenty-three additional therapeutic agents have been approved for use in humans [1]. The first drugs approved in the United States to treat HIV-1 infection inhibit the specific activity of the virally encoded reverse transcriptase, the viral enzyme essential for conversion of the viral RNA genome into a DNA provirus that integrates itself into the host genome. Two classes of reverse transcriptase inhibitors are currently marketed—nonnucleoside reverse transcriptase inhibitors (NNRTIs) and nucleoside/nucleotide reverse transcriptase inhibitors (N(t)RTIs) [2]. Another approved and marketed class of HIV-1 antiviral therapeutics inhibits the HIV-1 protease, a viral enzyme required to process newly synthesized viral polyproteins into the mature viral gene products, enabling the virus to assemble itself into new infectious virus particles [3]. A third class of HIV-1 therapeutics inhibits viral infection by preventing virus attachment to the host cell CCR5 chemokine receptor or prevents the fusion of the viral and cellular membranes [4]. Most recently, compounds which prevent the integration of the HIV-1 proviral precursor into cellular DNA have been successfully developed and utilized. Clinical experience with all HIV-1 agents has clearly demonstrated the ability of HIV-1 to easily evade the antiviral effects of any monotherapeutic drug administration strategy through the rapid accumulation of amino acid changes in the targeted proteins—reverse transcriptase, protease, envelope, and integrase [5]. The high turnover rate of virus replication along with the highly error prone HIV-1 reverse transcriptase, with its lack of proofreading capability, generates significant heterogeneity within the highly related but nonidentical populations (or quasispecies) of viruses circulating in a patient [6]. It is widely accepted that most drug-resistant viruses preexist within the population of viruses and are selected from within this heterogeneous environment upon application of selective drug pressure [7]. In addition to the high levels of resistance possible to single agents, each of the anti-HIV-1 agents employed to date has had significant dose limiting and long-term toxicities that render successful long term therapy for HIV-1 disease difficult to achieve [8]. 

In much of the developing world, antiretroviral therapy has successfully suppressed HIV-1 replication in patients, allowing significant delays to the progression of AIDS and in some cases completely normal life spans. However, HIV-1 therapies in general are plagued by patient compliance issues reflective of difficult treatment regimens, involving up to four antiretroviral drugs, significant class-specific toxicity [9], and the emergence and spread of virus isolates selected for resistance to single or multiple antiretroviral agents [10]. In the developing world many of these therapeutic strategies are uniformly unavailable due to the prohibitive cost of the drugs. The absence of an effective vaccine and the lack of effective therapy means that sub-Saharan Africa and Southeast Asia, among other developing regions of the world, remain epicenters for the continued spread of HIV-1, especially among heterosexual women [11]. In these areas of extremely high HIV-1 transmission rates, the opportunities to derail the AIDS pandemic rest on the processes of education and the development of effective topical microbicides, a specific HIV-1 prevention strategy employing HIV-1 drugs to prevent the sexual transmission of HIV-1 [12]. 

## 2. Identification and IND-Directed Development of New Antiretroviral Agents

The FDA has published guidance documents that relate to the development of systemic HIV-1 inhibitors [1]. These documents define the preclinical pharmacologic data that must be provided in an IND submission to begin human testing of a new antiretroviral agent. The submitted data package must specifically address the efficacy and toxicity of the test compound in a relevant cell-based assay system. In addition studies should be initiated that adequately address the range and mechanism of action of the test compound. With the wide variety of approved anti-HIV-1 drugs already on the market and the demonstrated efficacy of highly active antiretroviral therapies (HAARTs) [13], the ability of test compounds to be utilized as a component of combination drug therapies with the approved HIV-1 drugs should be evaluated in detail. Finally, drug resistance should be evaluated to define the ease of selection of resistant strains and to define diagnostic resistance-engendering mutations prior to clinical trials. Animal models to evaluate the effectiveness of HIV-1 therapies are available but their predictability for clinical efficacy is still highly debated, and thus most drug development programs bypass these animal models and move directly to Phase I human safety trials.

It is clear that highly active antiretroviral therapy (HAART) has significantly decreased morbidity and mortality among patients infected with HIV-1 and has prolonged the life of infected individuals. HAART has transformed HIV-1 infection from a lethal infection to a chronic disease much like diabetes. However, new anti-HIV-1 agents are still needed to confront the emergence of drug resistance and various adverse effects associated with long-term use of antiretroviral therapy. New antiviral agents that inhibit an increasing number of viral and cellular processes are critical for treating infected patients, as well as for prophylactic use, and all possible targets for countering HIV-1 infection, replication, and persistence need to be considered. Finally, efforts to eradicate HIV-1 from latent reservoirs within the body have gained increasing traction with the success of HAARTs and eradication efforts will require novel drugs and new ways of thinking about antiretroviral therapy of latent and silent HIV-1 infection. 

The identification of new antiretroviral agents typically involves either cell-based or biochemical/enzymatic target-based screening programs. The end result of these screening programs is a lead compound which provides a pharmacophore for medicinal chemistry structure-activity relationships (SARs) efforts to enhance the potency (increased efficacy and/or reduced toxicity) of the lead molecule, subsequently yielding candidates which progress through the IND-directed clinical development pathway to human clinical trials. Historically, new drug entities have been highly specific virus-targeted agents which inhibited critical steps in HIV-1 replication. Current development efforts continue to exploit the known targets for antiretroviral intervention, but have been expanded to include agents which target cellular processes that are essential for HIV-1 replication. Genomic, proteomic, and metabolomics approaches have identified large numbers of cellular products and pathways that are positively and negatively impacted by HIV infection, providing a large number of potential novel anti-HIV targets [14]. Herein we provide an overview of current and continuing drug development in the HIV-1 field based on the target of the antiretroviral agent as well as an overview of the methodology utilized to identify and confirm the new molecules as potential drug candidates for clinical development. Methods available to identify and characterize the mechanism of action of new antiretroviral agents are summarized in Table 1.

## 3. HIV-1 Entry Inhibitors

Though a range of hematopoietic cells, including monocyte-macrophages, B lymphocytes, eosinophils, and dendritic cells, as well as columnar epithelial cells, have been found to be infected by HIV-1, the CD4-positive helper T lymphocyte has been identified as the primary target for HIV-1 infection [15]. HIV-1 enters CD4-positive T cells through direct interaction of the viral envelope gp120 with the D1 region of the CD4 receptor on the cell surface of target cells. The interaction of gp120 with CD4 causes a conformational change in the viral envelope gp120, resulting in exposure of the gp41 transmembrane envelope protein which subsequently inserts into and fuses with the target cell membrane. HIV-1 envelope proteins interact with coreceptor molecules on the surface of CD4-positive cells, typically either the *α*-chemokine receptor CXCR4 or the *β*-chemokine receptor CCR5 or both, to trigger the fusion of the viral and cellular membranes. The HIV-1 fusion inhibitor, enfuvirtide, developed by Hoffmann-La Roche and Trimeris, was the first therapeutic in its class to be approved for use in humans by the FDA [30]. Enfuvirtide binds to gp41 and prevents the pore formation required for the capsid of the virus to enter the target cell. Since enfuvirtide is a peptide, the drug is marketed in an injectable form, which has somewhat limited its therapeutic utility. In addition, primary resistance mutations in the HR1 region of gp41 have been identified in 10.5% of enfuvirtide-naïve patients which allow the virus to evade the antiviral effects of the drug [31]. The CCR5 coreceptor antagonist, maraviroc, was developed by Pfizer, and the FDA approved maraviroc for combination therapy in 2007 [32]. By blocking the HIV-1 gp120 protein from associating with the CCR5 coreceptor, maraviroc prevents HIV-1 from entering the target cell. However, since HIV-1 can use other coreceptors for entry, an HIV-1 tropism test must be performed to determine if the drug will be effective in a particular patient. CCR5-tropic HIV-1 strains are more common than CXCR4-tropic strains and have been identified as the strain which is predominantly transmitted, suggesting that maraviroc will be useful for both prevention of virus transmission (topical microbicide use) and treatment. Additionally, the CXCR4 coreceptor is more critical for immune function and cannot be safely blocked, indicating that CXCR4-targeted inhibitors would be immune-toxic in the host. Among individuals found mostly in Northern Europe, there is a polymorphism in CCR5, involving a 32-base pair inactivating deletion known as delta32 (Δ32), which reduces or completely eliminates cell surface expression of CCR5 [33]. Individuals with one CCR5-Δ32 polymorphism exhibit reduced disease progression, while those homozygous for the deletion appear to have natural resistance to HIV-1 infection [33]. There appears to be no obvious immunologic detriment from the Δ32 deletion, making CCR5 a highly credible antiviral target. Other CCR5 antagonists completed or currently in Phase II clinical development include INCB9471 (Incyte), HGS004 (Human Genome Sciences), PRO140 (Progenics), PF232798 (ViiV Healthcare), cenicriviroc (Tobira Therapeutics), and VCH286 (ViroChem Pharma). CCR5 antagonists in Phase I clinical development include AK602 (Kumamoto University), SCH532706 (Schering), GSK706769 (ViiV Healthcare), and VIR576 (Viro Pharmaceuticals). Other entry inhibitors in development include SP01A (Samaritan Pharmaceuticals) in Phase III clinical trials and ibalizumab (Taimed Biologics), a nonimmunosuppressive monoclonal antibody that binds CD4 [34], in Phase II studies. HIV-1 can enter and bud from lipid rafts of plasma membranes of infected cells. Lipid rafts play a crucial role in colocalizing CD4 and chemokine receptors for entry of HIV-1 into T cells. Depletion of plasma membrane cholesterol relocalizes raft-resident markers to a nonraft environment and inhibits productive infection by HIV-1 [35]. SP01A affects cholesterol synthesis by reducing 3-hydroxy-3-methylglutary coenzyme A (HMG-CoA) reductase mRNA expression. Inhibition of cholesterol synthesis by SP01A modifies the cholesterol content of the host cell membrane lipid rafts and prevents HIV-1 fusion with CD4-positive cells. SP10A is a second generation oral entry inhibitor that is being developed by Samaritan Pharmaceuticals [35].

A number of preclinical assays have been developed to identify potential inhibitors of HIV-1 entry utilizing both replication competent wild type viruses and pseudotype viruses. Compounds may be evaluated for inhibitory activity in CD4-dependent and CD4-independent virus transmission assays. A variety of cell lines have been made which stably express CD4, CCR5, or both coreceptors on the cell surface to evaluate coreceptor inhibitors of HIV-1 infection. Evaluations can be performed to define the specific mechanism of antiviral action of compounds which are directly virucidal, or which inhibit virus attachment, or virus-cell fusion, or virus entry to the target cell using indicator cell lines with reporter gene endpoints (colorimetric, chemiluminescent, and fluorescent) to measure virus replication [16]. Compounds which directly interfere with binding of gp120 to CD4 can be evaluated via ELISA using purified proteins [17]. The effect of inhibitors on the virus gp120/CD4/coreceptor complex that would target gp41 can be evaluated using an indicator cell line, such as HeLa-LTR-CD4-*β*-galactosidase cells which employ a tat protein-induced transactivation of the reporter gene driven by the HIV-1 long terminal repeat promoter, and manipulating the fusion step with temperature changes. Varying the time of drug addition in high multiplicity of infection (MOI), single round of infection anti-HIV-1 assays is often useful in demonstrating that entry inhibitors must be present prior to 2 hours post-infection of target cells in order to provide antiviral activity. 

## 4. HIV-1 Reverse Transcriptase Inhibitors 

Inhibitors of HIV-1 reverse transcriptase can be divided into two classes: nucleoside/nucleotide reverse transcriptase inhibitors (NRTI/NtRTIs) and nonnucleoside reverse transcriptase inhibitors (NNRTIs). NRTIs are analogues of the naturally occurring deoxynucleotides required for synthesis of viral DNA and following phosphorylation to the active triphosphate form by cellular kinases; they compete with the natural deoxynucleotides for incorporation into the growing viral DNA chain. However, unlike the natural deoxynucleotide substrates, NRTIs and NtRTIs lack a 3′-hydroxyl group on the deoxyribose moiety or are pseudosugars unable to be extended. As a result, following incorporation of an NRTI or an NtRTI, the next incoming deoxynucleotide cannot form the 5′-3′ phosphodiester bond needed to extend the DNA chain and thus causing chain termination. Mitochondrial toxicity is recognized as a major adverse effect of nucleoside analogue treatment. Nucleoside analogues are effective in inhibiting HIV-1 replication due to their high affinity for the viral RT enzyme. However, NRTIs can also bind to other human DNA polymerases, like DNA polymerase beta, necessary for repair of nuclear DNA, and mitochondrial DNA polymerase gamma, which is exclusively responsible for the replication of mitochondrial DNA. NRTIs and NtRTIs comprise the first class of antiretroviral drugs developed and approved for use in humans to treat HIV-1 infection. There are a number of FDA-approved nucleoside/nucleotide reverse transcriptase inhibitors. Cytidine analogs include zalcitabine (ddC), which is no longer marketed, emtricitabine (FTC), and lamivudine (3TC). Thymidine analogs include zidovudine (AZT) and stavudine (d4T). Didanosine (ddI) and tenofovir disoproxil fumarate (TDF) are analogs of adenosine, and abacavir sulfate (ABC) is an analog of guanine. HIV-1 can become resistant to NRTIs by two mechanisms. The first resistance mechanism involves the reduced incorporation of the nucleotide analog into DNA over the normal nucleotide. This resistance mechanism results from mutations in the N-terminal polymerase domain of the reverse transcriptase that reduce the enzyme's affinity or ability to bind to the drug. A prime example of this mechanism is the M184V mutation that confers resistance to lamivudine (3TC) and emtricitabine (FTC). Another well-characterized set of mutations is the Q151M complex found in multidrug-resistant HIV-1 which decreases reverse transcriptase's efficiency at incorporating NRTIs but does not affect natural nucleotide incorporation. The complex includes the Q151M mutation along with amino acid changes A62V, V75I, F77L, and F116Y. A virus with Q151M alone is moderately resistant to zidovudine (AZT), didanosine (ddI), zalcitabine (ddC), stavudine (d4T), and slightly resistant to abacavir (ABC). A virus with Q151M in concert with one or more of the other four noted mutations becomes highly resistant to those drugs and is additionally resistant to lamivudine (3TC) and emtricitabine (FTC) [36]. A virus with the Q151M complex in addition to the K70Q mutation significantly enhanced resistance to several approved NRTIs and also resulted in 10-fold resistance to TDF [37]. The K65R mutation emerges in response to treatment with TDF, ABC, ddI, or d4T and has been shown to have an increased frequency in subtype C HIV-1 [38]. The second resistance mechanism involves the ATP-based excision of the incorporated drug by 3′→5′ exonuclease activity, which allows the DNA chain to be extended and polymerization to continue [39]. Excision enhancement mutations, typically M41L, D67N, K70R, L210W, T215Y/F, and K219E/Q, are selected by thymidine analogs AZT and D4T and are therefore referred to as thymidine analog mutations (TAMs). The excision-based mutations improve the ability of the RT to bind ATP. ATP-dependent pyrophosphorylation removes the drug and releases a dinucleotide tetraphosphate. The goal of next generation reverse transcriptase inhibitors is to treat patients with multidrug-resistant HIV-1, prolong the time to emergence of drug resistance to the new inhibitors, and to increase drug adherence by minimizing pill burden and side effects. Several NRTIs are in development to treat HIV-1-infected patients. Entecavir (ETV), a guanine analog for HIV-1 infection, is currently in development by Bristol-Myers Squibb and has been FDA approved for treatment of HBV infection since 2005. Apricitabine (ATC), a cytidine analog with antiviral activity against 3TC and AZT-resistant HIV-1 being developed by Avexa Pharmaceuticals, was given fast track approval by the FDA in March 2011. Dexelvucitabine (DFC) and racivir are cytidine analogs in development by Pharmasset. DFC is active against drug-resistant HIV-1 containing the M184V, K65R, L74V and TAMS mutations. However, Incyte discontinued co-development of DFC due to increased incidence of grade 4 hyperlipasemia, a marker of pancreatic inflammation, in a Phase IIb clinical trial. Racivir has completed a Phase II clinical trial in comparison with lamivudine in patients with the M184V lamivudine-resistant virus. Elvucitabine is a cytosine nucleoside analog of stavudine which was evaluated in a Phase II clinical trial by Achillion Pharmaceuticals. Unimpressive clinical results did not provide a rationale for further development of the drug. Another derivative of stavudine, festinavir, is being developed by Bristol-Myers Squibb and has antiviral activity against multidrug-resistant HIV-1 with less toxicity compared to stavudine. Chimerix, Inc. developed a lipid conjugate of tenofovir and unlike tenofovir, disoproxil fumarate and most prodrugs, the CMX157 prodrug is not efficiently cleaved in plasma thus increasing the levels of active tenofovir in target cells. CMX157 is greater than 300 times more potent than tenofovir with increased oral bioavailability [40]. Following a favorable Phase I clinical trial, Chimerix is seeking to outlicense the compound for further development. Another prodrug of tenofovir, GS-7340, is being developed by Gilead Sciences to better target lymphoid tissues and cells [41]. GS-7340 has increased plasma stability compared with tenofovir. A recent Phase I clinical trial resulted in no serious adverse effects. Investigators at the University of Georgia identified 1-(*β*-D-dioxolane) thymine (DOT) as a potent inhibitor of AZT- and 3TC-resistant HIV-1 strains, and this compound is currently in a Phase I clinical trial [42]. Medivir is developing MIV-210, a nucleoside analog with potent antiviral activity versus drug resistant HIV-1 as well as hepatitis B virus. Following favorable plasma levels of MIV210 and good oral bioavailability in Phase I studies, a Phase IIa clinical trial has been initiated with multi-drug-resistant HIV-1 infected patients.

In contrast, to the NRTIs and NtRTIs, NNRTIs have a completely different mode of action. NNRTIs allosterically block reverse transcriptase by binding at a different site on the enzyme as compared to the chain terminating analogs. NNRTIs are not incorporated into the viral DNA but instead inhibit the movement of protein domains of reverse transcriptase essential for DNA synthesis. Since the hydrophobic binding area found in HIV-1 reverse transcriptase does not appear in HIV-2, NNRTIs are specific to inhibition of HIV-1 replication. NNRTIs do not bind to the active site of the polymerase but bind to a less conserved area near the active site in the p66 subdomain. NNRTI binding results in a conformational change in the reverse transcriptase that distorts the positioning of the residues that bind DNA, inhibiting polymerization. NNRTI resistance is conferred by mutations that decrease the binding of the drug to this pocket. Treatment with a regimen including efavirenz (EFV) and nevirapine (NVP) typically results in the appearance of mutations L100I, Y181C/I, K103N, V106A/M, V108I, Y188C/H/L, and G190A/S. Current FDA-approved NNRTIs also include delavirdine (Pfizer) and three diarylpyrimidines developed by Tibotec Therapeutics, dapivirine, etravirine and rilpivirine. The second-generation NNRTIs by Tibotec have better potency, longer half-life, and reduced side effects compared with the older NNRTIs, such as efavirenz. Delavirdine is not recommended for use as part of initial therapy due to its lower efficacy compared to other NNRTIs, interactions with other medications due to its inhibition of CYP3A4, and higher pill burden. As patients live longer on HAART and the pool of NNRTI-resistant virus increases, so does the need for the development of new NNRTIs with antiviral activity against both wild-type and the clinically prevalent NNRTI-resistant HIV-1 strains. Boehringer Ingelheim has presented data on BILR355BS, a dipyridodiazepinone NNRTI compound, with potent antiviral activity (EC_50_ < 10 nM) against a wide range of NNRTI-resistant viruses but terminated drug development during the Phase II clinical trial [43]. GSK2248761, belonging to the family of 3-phosphindoles, was developed by ViiV Healthcare and completed Phase II studies, but the FDA put further development on hold due to significant adverse events (seizures). It is unclear if or when development will continue. RDEA806, a new family of triazole NNRTIs, entered Phase IIb clinical trials by Ardea Biosciences in 2009. Lersivirine, developed by Pfizer, belongs to the pyrazole family and completed Phase IIb studies in 2010. The resistance profile for compounds in development is similar to that of other next generation NNRTIs. ImQuest Pharmaceuticals has recently reported a pyrimidinedione NNRTI with highly potent anti-HIV-1 activity and a dual mechanism of action which also involves the inhibition of virus entry [18]. Their lead compound (IQP-0528) is expected to soon enter human clinical trials for both therapeutic and topical microbicide use.

Inhibition of the virus-encoded reverse transcriptase can be evaluated in both cell-based and biochemical assays. High MOI and time of drug addition anti-HIV-1 assays are often useful in demonstrating that RT inhibitors must be present prior to 8 hours postinfection of target cells in order to yield antiviral activity. In cell-based assays, PCR amplification of early, intermediate, and late RT products may be analyzed in treated, HIV-1-infected cells to determine inhibition of enzymatic activity compared to an untreated, infected cell culture. A biochemical assay utilizing purified, recombinant RT enzymes can also be used to identify inhibitors of wild type and drug-resistant HIV-1 reverse transcriptase [19].

## 5. HIV-1 RNase H Inhibitors 

The ribonuclease H (RNase H) function of the C terminus of reverse transcriptase is required for successful production of a DNA copy of the HIV-1 genome. RNase H is required for processing the tRNA primer used to begin minus-strand DNA synthesis and degradation of the viral RNA during synthesis, followed by preparation of the polypurine tract (PPT) DNA-RNA hybrid, which serves as the primer for positive-strand DNA synthesis. Essential for RNase H activity is a group of three carboxylate-containing amino acid residues, conserved in the class of polynucleotidyl transferases and a fourth conserved in RNase H [44]. RT-RNase H is absolutely essential for HIV-1 replication and is therefore a logical and thus far unexploited target for antiretroviral intervention. Drug discovery efforts focusing on RT-RNase H have lagged behind those for other HIV-1 targets but are ongoing. Nucleoside and nonnucleoside compounds have been reported to inhibit both the polymerase and RNase H activities, though the mechanism of RNase H inhibition is poorly understood. Studies have shown that the NRTI AZT and the NNRTI EFV act in a synergistic fashion (together they inhibit RT function to a greater extent than the sum of their individual inhibitory activities). It has been demonstrated that RT inhibition by EFV may allow the innate RNAse H activity of RT to cleave the RNA template, which, in turn, increases susceptibility to AZT, yielding a synergistic antiviral interaction of the two drugs [45]. AZT incorporates into the growing DNA chain, stopping reverse transcription unless it is excised. In the presence of EFV, RNase H activity of RT is enhanced, leading to destruction of the RNA template before AZT excision can efficiently occur, increasing the apparent activity of AZT [46]. An obstacle to the development of RNase H inhibitors was highlighted in a study of *β*-thujaplicinol [47] that measured cleavage of RNA by RNAse H. *β*-thujaplicinol efficiently cleaved RNA strands; however, in the context of reverse transcriptase tightly bound to the RNA substrate, the conformational change during reverse transcription resulted in *β*-thujaplicinol being unable to inhibit RNase H. This suggested that RNase H inhibitors, such as *β*-thujaplicinol and dihydroxyl benzoyl naphthyl hydrazine (DHBNH) [47], that bind directly to the RNase H active site within RT might have difficulty accessing this site during transcription when RT is bound to an RNA template. RNase H inhibitors that do not bind in the active site of RNase H within HIV-1 RT, such as the MK3 naphthyridine [48], should be explored, and potential antagonism with other RT inhibitors will need to be addressed. RNase H proteins are native to all forms of life, so building inhibitor specificity toward HIV-1 RNase H without off-target effects will be critical to developing an effective drug.

Inhibitors of RNase H function have been identified using a biochemical polymerase-independent cleavage assay with a 5′-[^32^P]tC5U/p12 substrate [20]. The radioactive RNA-DNA chimera is hybridized to its DNA complement, which mimics processing of the HIV-1-1 PPT primer from nascent DNA, following initiation of second-strand synthesis. Capillary electrophoresis is used to illustrate RNase H cleavage at the PPT RNA-U3 DNA junction and at two additional positions.

## 6. HIV-1 NCp7 Inhibitors 

HIV-1 p7 nucleocapsid protein (NCp7), which contains two highly conserved zinc fingers with a nonclassical Cys-Xaa2-Cys-Xaa4-His-Xaa4-Cys (CHHC) sequence, is a maturational proteolytic product of the p55 precursor polyprotein [49]. The zinc fingers function in selection and incorporation of viral RNA into budding virions while being a component of the p55 precursor. Zinc fingers of the NCp7 are required for the initial infection of target cells, promote initiation of transcription, and increase the efficiency of template switching during reverse transcription. Due to the essential and pluripotent roles in both early and late stages of HIV-1 replication, as well as the conserved Cys and His chelating residues, the HIV-1 zinc fingers represent attractive antiviral targets and would appear to be multifunctional inhibitors of HIV-1. Disulfide-substituted benzamides (DIBAs) were identified as anti-HIV-1 inhibitors with the ability to chemically modify and eject zinc from the zinc finger of NCp7. Antiviral activity of the DIBAs resulted in the formation of noninfectious virus or in the complete inhibition of virus production *in vitro*, similar to HIV-1 protease inhibitors. Azodicarbonamide (ADA; HPH116) is a nucleocapsid inhibitor that electrophilically attacks the sulfur atoms of the zinc-coordinating cysteine residues of the CCHC domain [50]. ADA is directly virucidal by preventing the initiation of reverse transcription and blocking formation of infectious virus by modification of the CCHC domain within Gag precursors. ADA was evaluated in a Phase II study in 2001, but the status of drug development is unknown. S-acyl-2-mercaptobenzamide thioesters (SAMTs) demonstrate potent antiviral activity *in vitro* as a virucidal agent and in *in vivo* SIV studies in Cynomolgus macaques [51]. NV038, a N,N′-bis(1,2,2-thiadiazol-5-yl)benzene-1,2-diamine, targets NCp7 by reacting with the sulfhydryl group of cysteine residues. NV038 acts via a different mechanism than other reported zinc ejectors, as its structural features do not allow an acyl transfer to Cys or a thiol-sulfide interchange [52]. Studies performed at ImQuest BioSciences have demonstrated a significant inability to select for drug resistant viruses to the zinc finger inhibitors as well as their highly synergistic interaction with all classes of antiretroviral agents.

NCp7-targeted inhibitors have been shown to be virucidal *in vitro*. Cell-free virus is treated with compound then washed away prior to incubation with target cells to demonstrate reduction in virus infectivity [51]. In addition, the zinc finger inhibitors reduce virus production from chronically HIV-1-infected cells. Zinc ejection from purified NCp7 protein can also be assessed biochemically in the presence of inhibitors [52]. Specificity of NCp7 inhibition for the retroviral zinc finger should be addressed by evaluating the interaction of inhibitors with cellular Sp1, GATA, and PARP zinc fingers. 

## 7. HIV-1 Integrase Inhibitors 

Integrase is a viral enzyme that integrates retroviral DNA into the host cell genome. HIV-1 integration occurs through a multistep process that includes two catalytic reactions: 3′ endonucleolytic processing of proviral DNA ends and integration of 3′-processed viral DNA into cellular DNA, referred to as strand transfer. The 3′ processing integrase binds to a short sequence located at either end of the long terminal repeat (LTR) of the viral DNA and catalyzes endonucleotide cleavage, resulting in elimination of a dinucleotide from each of the 3′ ends of the LTR. Cleaved DNA is then used as a substrate for integration. Strand transfer occurs simultaneously at both ends of the viral DNA molecule, with an offset of five base pairs between the two opposite points of insertion. Integration is completed by removal of the unpaired dinucleotides, repair of the single-stranded gaps created between the viral and target DNA, and ligation of the host DNA. Divalent metals, Mg^2+^ or Mn^2+^, are cofactors required for 3′-processing and strand transfer steps. Raltegravir, the first integrase inhibitor developed by Merck Sharp & Dohme Limited, was FDA approved for use in HIV-1-infected patients in 2007. Other HIV-1 integrase inhibitors currently in Phase III clinical trials include elvitegravir, developed by Japan Tobacco, and dolutegravir, developed jointly by ViiV Healthcare and Shinongi. Raltegravir and elvitegravir possess metal-chelating functions and interact with divalent metals within the active site of HIV-1 integrase. The inhibitors compete directly with viral DNA for binding to the integrase active site at the DDE motif, a highly conserved triad of acidic residues consisting of D64, D116, and E152 which mediate binding of the metal cofactors to the active site, in order to block strand transfer [53]. Two structural components are necessary for integrase binding: a hydrophobic benzyl moiety that buries into a highly hydrophobic pocket near the active site and a chelating triad that binds with two Mg^2+^ ions in a hydrophilic region, anchoring the inhibitor onto the protein surface. Identification of the pharmacophore for inhibition of HIV-1 integrase catalysis has proven to be challenging. For optimal integrase inhibition, the pharmacophore requires a region-specific (N-1) diketoacid of specific length [54]; however, a detailed binding model is lacking, so it has been difficult to develop structure-based design of integrase inhibitors. HIV-1 resistance to raltegravir and elvitegravir has been associated with mutations in the loop of amino acid residues 140–149. Raltegravir has limited intestinal absorption, and thus resistance cannot be overcome by prescribing higher doses. The integrase inhibitor dolutegravir is sensitive to HIV-1 variants resistant to raltegravir or elvitegravir, is bioavailable as a single, oral dose without need of a booster, and has been well tolerated by patients in clinical trials. Clinical trials are underway to support the use of dolutegravir in combination with abacavir and lamivudine, in a new fixed dose combination called 572-Trii. GSK1265744 is in Phase IIa human clinical trials as a new generation candidate to dolutegravir. Merck has developed a second generation integrase inhibitor, MK-2048, with the same mechanism of action as raltegravir with sensitivity to raltegravir-resistant HIV-1 [55]. MK-2048 is being investigated for use as part of preexposure prophylaxis (PrEP) regimen and has been shown to inhibit the integrase enzyme four times longer than raltegravir. BI224436 is in preclinical development by Gilead Sciences following its purchase from Boehringer Ingleheim as a novel noncatalytic site integrase inhibitor that binds to a conserved allosteric pocket of the HIV-1 integrase enzyme [56]. BI224436 has been shown to retain full antiviral activity against viruses encoding resistance mutations to clinically approved drugs targeting HIV-1 integrase. BI224436 has advanced to Phase I clinical trials following ADME evaluations which indicated favorable metabolic stability, low potential for interactions with CYP3A4 and CYP2D6, high permeability, excellent physicochemical properties, and excellent pharmacokinetic profiles in animals.

Structural studies utilizing cocrystallization with prototype foamy virus (PFV) intasome with raltegravir and elvitegravir have been helpful in establishing the binding mode of integrase strand transfer inhibitors. Crystal structures of PFV intasomes containing primary mutations associated with drug resistance, as well secondary amino acid substitutions which may compensate for the impaired viral fitness, revealed conformational rearrangements within the IN active site contributing to raltegravir resistance [21]. Integration of the 2-LTR circular cDNA into the host DNA mediated by the virus-encoded integrase can be evaluated for inhibition in both cell-based and biochemical assays. In a high MOI single-round HIV-1 infection in cells, PCR detection of the provirus in genomic DNA can be assessed. Amersham produces an HIV-1 integrase scintillation proximity assay (SPA) enzyme kit for biochemical evaluation of potential integrase inhibitors [23]. An *in vitro* assay utilizing integrase-mutant HIV-1 molecular clones complemented in trans by Vpr-IN fusion proteins enabled the study of integrase function in replicating viruses [22]. 

## 8. HIV-1 Regulatory and Accessory Protein Inhibitors

After integration into the host genome, HIV-1 remains quiescent until basal transcription produces a threshold level of the viral transactivator protein, Tat. Tat increases viral mRNA production several hundredfold by increasing the elongation capacity of RNA polymerase II (Pol II) rather than initiation of transcription. Tat is brought into contact with the transcription machinery after binding the transactivation-responsive (TAR) element, a 59-residue stem loop RNA found at the 5′ end of all HIV-1 transcripts. Tat forms a tight, specific complex with TAR RNA centered on a U-rich region found near the apex of the TAR RNA stem. Interactions between Tat and TAR are absolutely required for the increased processivity of Pol II and the production of full length virus transcripts. Tat binds to the cyclin-dependent kinase 7 (CDK7) and activates the phosphorylation of the carboxy-terminal domain of Pol II by TFIIH and the CDK-activating kinase (CAK) complex [57]. Studies suggest the interaction between Tat and its cellular counterpart is critical for the function of Tat and the increased processivity of Pol II. Oligonucleotides have been investigated for inhibition of Tat binding to this recognition site in biochemical assays, but they failed to disrupt HIV-1 replication in acute infection of primary lymphocytes [58]. Natural 4-phenylcoumarins isolated from *Marila pluricostata* were identified as Tat antagonists and were able to inhibit HIV-1 replication in cell-based assays [24]. Based on the beta-turn motif present in HIV-1 Tat, a series of novel benzodiazepine analogs were designed as biological mimetics. Preliminary biological evaluation exhibited inhibitory activity on HIV-1 Tat-mediated LTR transcription [59]. BPRHIV001, a coumarin derivative, has been identified as an HIV-1 Tat transactivation inhibitor (EC_50_ of 1.3 nM) with synergistic effects in combination with currently used reverse transcriptase inhibitors [60].

The Rev protein is an essential factor for HIV-1 replication and promotes the export of unspliced or partially spliced mRNA responsible for the production of the viral structural proteins. Within the N-terminal of Rev is the arginine-rich motif (ARM) which comprises both the nuclear localization signal (NLS) to mediate the nuclear and nucleolar localization of Rev and the RNA-binding domain to mediate binding of Rev to the Rev-Responsive Element (RRE), a 240-base region of complex RNA secondary structure. Flanking the ARM are sequences involved in mediating Rev multimerization that appears to be critical for its biological role. Polymerized Rev that interacts with host cellular factors is a prerequisite for RNA binding. The interaction between the HIV-1 Rev protein and the RRE RNA is an attractive target for antiviral therapy due to its role in facilitating the nuclear export of incompletely processed viral transcripts and its necessity for viral replication. For HIV-1, targeting the host cell factors might elicit fewer drug-resistant viruses. Screening for Rev inhibitors is in the early preclinical drug development stage, and various researchers have targeted the nuclear export factor CRM1, interference with the Rev-RRE interaction, Rev protein itself, and other cellular factors involved in HIV-1 transcription [26]. Leptomycin B (LMB), a *Streptomyces* cytotoxin discovered as a potent antifungal antibiotic that blocks the eukaryotic cell cycle, binds CRM1 and disrupts NES-mediated nuclear transport [61]. Variability in LMB production lots in *Streptomyces* cultures that vary greatly in toxicity has hampered the use of LMB. PKF050-638 is also capable of blocking Rev function by binding to CRM1 at position Cys-539 but its cellular toxicity resulted in the failure to pursue its potential as a therapeutic [62]. Neomycin B is capable of interfering with the Rev-RRE interaction, but poor efficacy (EC_50_ of 2.5 mM), toxicity, and poor oral absorption have prevented its development as a useful antiviral drug [63]. Diphenylfuran cations have also been shown to interfere with the Rev-RRE interaction *in vitro* at 0.1 *μ*M concentrations. These aromatic cationic compounds bind tightly to the minor groove of the IIB Rev motif with pronounced selectivity [64]. Antisense oligonucleotides which interact with RRE-IIB have also been investigated and found to bind with specificity and high affinity with apparent dissociation constants in the nanomolar range [65]. Thiabendazole, chlorpropham, and a series of related analogs which inhibit HIV-1 at a late stage, postintegration step of virus replication were identified by The Proctor & Gamble Company and are being investigated by ImQuest BioSciences. The compounds were identified as inhibitors of HIV-1 replication from chronically HIV-1-infected cells with the ability to suppress constitutive virus production in the long term. Mechanistic studies indicate the treatment of infected cells with these compounds results in an accumulation of multiply spliced viral RNA, with a corresponding decrease in the quantity of singly spliced and unspliced viral RNA, suggesting the compounds may inhibit Rev function. 

A novel mechanism of antiviral action recently exploited by Trana Discovery involves human transfer RNA (tRNA) as a therapeutic target. The role of tRNA^Lys3^_SUU_ is essential for the replication and survival of HIV-1 at both reverse transcription as a primer and virus assembly, thereby providing a dual point of intervention by tRNA inhibitors. Efforts to inhibit the tRNA^Lys3^_SUU_ have centered on mimicking the anticodon stem loop (ASL) of tRNA to prevent binding of viral RNA [66]. 

Nef is a multifunctional accessory protein of HIV-1 which is critical for high virus replication and disease progression in infected patients. The lack of disease progression in patients infected with* nef*-deleted HIV-1, such as the Sydney Blood Bank Cohort comprised of eight individuals infected with an attenuated, nef/LTR-deleted strain of HIV-1 from a single donor, defines Nef as a pathogenic factor [67]. Developing inhibitors of Nef in order to reduce the severity of HIV-1 disease has been difficult due to the complexity of Nef's multiple functions. Nef is a small protein devoid of enzymatic activity that serves as an adaptor protein to divert host cell proteins to aberrant functions that amplify viral replication. Investigation of Nef function has led to the possibility of developing new anti-HIV-1 drugs targeting Nef's ability to induce CD4 downmodulation, major histocompatibility complex I and II (MHCI/MHCII) downmodulation, Pak2 activation, inhibition of p53 and ASK-1 involved in apoptosis, and enhancement of virion infectivity. Nef-induced CD4 downmodulation involves the internalization of surface CD4 followed by degradation via the endosomal/lysosomal pathway. Inhibition of lysosomal acidification blocks Nef-induced CD4 degradation, without restoring CD4 surface expression. The clathrin-associated adaptor protein 2 (AP2) is a key molecular mediator of Nef-induced CD4 downmodulation, suggesting this interaction is a possible target for antiviral therapy [68]. Another well-conserved property of Nef is its ability to downmodulate MHC class I molecules that enables the infected cell to evade destruction by the immune system during active viral replication. A ternary complex between the cytoplasmic tail of MHC and AP1, with Nef acting as a facilitator, may activate a tyrosine sorting signal in the MHC which diverts newly synthesized MHC molecules from their transit to the plasma membrane to an internal compartment. This ternary complex engages Nef in a novel interaction and could be a potential target for an antiviral compound. Nef may regulate cellular activation through several kinases, such as Pak2 and Hck. Nef binding with Pak2 has been demonstrated to activate Pak2 in multiple HIV-1 subtypes. However, the structural fluidity of Nef's Pak2 interaction surface could make this Nef interaction difficult to target with antiviral compounds. Structure-function analyses identified an SH3 domain interaction of Hck that interacts with Nef. A series of small Nef interacting proteins composed of an SH3 domain fused to a sequence motif of the CD4 cytoplasmic tail and a prenylation signal for membrane association were investigated [25] and identified two hydrophobic pockets on Nef as potent pharmacophore target sites. Nef augments the infectivity of HIV-1 particles and accounts for the slight delay in replication kinetics observed for* nef*-deficient HIV-1. Triciribine (TCN) is a tricyclic nucleoside that once phosphorylated to its 5′ monophosphate form by intracellular adenosine kinase is active against a wide range of HIV-1 and HIV-2 isolates. TCN was determined to be a late stage inhibitor of HIV-1 replication, and sequencing of TCN-resistant HIV-1 resulted in five-point mutations in the DNA sequence of *nef *[27]. Originally developed as an anticancer therapy, clinical trials indicated severe adverse toxicity with TCN such as hepatic toxicity, hyperglycemia, and thrombocytopenia [69]. Despite the attractiveness of a drug that reduces the inherent infectivity of HIV-1 virions, the prospects for inhibiting Nef-mediated enhancement of infectivity are remote. Overall attempts to develop inhibitors of Nef have demonstrated relatively low binding affinity, high cytotoxicity, and interference with only a subset of Nef interactions and functions. 

Viral protein U (Vpu) is a type 1 membrane-associated accessory protein encoded by HIV-1 and functions to form a virus ion channel. Vpu contributes to HIV-1-induced CD4 receptor downregulation by mediating the proteosomal degradation of newly synthesized CD4 in the endoplasmic reticulum. Vpu also enhances the release of progeny virions from infected cells by antagonizing tetherin, an interferon-regulated host restriction factor that directly cross-links virions on the host cell surface [70]. BIT225 was developed by Biotron Limited as a small molecule inhibitor of HIV-1 Vpu to specifically target HIV-1 in the monocyte-macrophage reservoir, similar to tetherin-mediated reduction in infectivity [71]. BIT225 is active against multiple drug-resistant strains of HIV-1, and Phase IIb clinical trials are currently in progress. 

Vpr is a multifunctional accessory protein critical for efficient viral infection of CD4-positive T cells and macrophages. Vpr mediates nuclear transport of the HIV-1 preintegration complex (PIC), induces G2 cell cycle arrest, modulates T-cell apoptosis, transcriptionally coactivates viral and host genes, and regulates nuclear factor kappa B (NF-*κ*B) activity [72]. The numerous functions of Vpr in the viral life cycle suggest that Vpr would be an attractive target for HIV-1 therapeutics. Di-tryptophan containing hexameric peptides have been reported to overcome Vpr-mediated cell growth arrest and apoptosis by interfering with nuclear translocation [73]. Damnacanthal (Dam), an anthraquinone derivative isolated from the Tahitian noni fruit, has been identified as an inhibitor of Vpr-induced cell growth cessation [74]. Vipirinin, a 3-phenyl coumarin-based compound in the RIKEN Natural Products Depository, inhibits Vpr-dependent viral infection of human macrophages. The hydrophobic region of residues Glu-25 and Gln-65 was found to be potentially involved in the binding of vipirinin to Vpr [75].

Viral infectivity factor (Vif) is a small, phosphoprotein essential for HIV-1 replication and pathogenesis. Vif neutralizes the host cell antiviral factor, apolipoprotein B mRNA editing enzyme catalytic polypeptide like 3G (APOBEC3G; A3G), which makes the viral particles more infective [76]. RN-18 was identified as an antagonist of Vif function and inhibited HIV-1 replication only in the presence of A3G. RN-18 increases cellular A3G levels in a Vif-dependent manner and increases A3G incorporation into virions without inhibiting general proteasome-mediated protein degradation in order to decrease virus replication [77].

The expression of HIV-1 regulatory proteins occurs early in the infected cell and is critical for appropriate replication of the virus. The ability of an anti-HIV-1 agent to inhibit these regulatory proteins can be evaluated in cell-based reporter assays, analyzed by Northern or Western blot, and by direct biochemical inhibition assays [24–27, 70, 73, 77].

## 9. Protease Inhibitors 

HIV-1 aspartyl protease is a C2-symmetric homodimer that catalyzes the proteolytic cleavage of the polypeptide precursors into mature enzymes and structural proteins. Inhibitors have been designed to mimic the transition state of the protease substrates. A peptide linkage consisting of –NH–CO– is replaced by a hydroxyethylene group, where the protease is unable to cleave. Mutations that confer resistance to HIV-1 protease inhibitors are located primarily in the active site of the enzyme that directly changes the binding of the inhibitor. Nonactive site mutations have been shown to alter dimer stability and conformational flexibility. Over 26 protease inhibitor-specific mutations have been described, of which 15 are primary mutations significant enough to reduce drug efficacy. High-level drug resistance typically requires multiple mutations in the HIV-1 protease. Often, these resistance-associated mutations reduce the catalytic efficiency of the protease, resulting in immature or noninfectious viruses. In addition, mutations develop within Gag cleavage sites, complementing the changes in the resistant protease. Significant associations have been observed between mutations in the nucleocapsid-p1 (NC-p1) and the p1-p6 cleavage sites and various mutations in protease associated with protease inhibitor resistance [78]. Gag A431V or the I437V mutation, within the NC-p1 cleavage site, has been associated with the V82A, I50L, or I84V protease mutations. Gag L449F/P, R452S, P453L mutations within the p1-p6 cleavage site have been associated with I50V or D30N/N88D protease mutations. Cross-resistance is one of the major problems of protease inhibitor treatment. FDA-approved protease inhibitors saquinavir (Hoffman-La Roche), ritonavir (Abbott Laboratories), and indinavir (Merck) are peptidomimetic compounds designed to fit the C2 symmetry in the protease-binding site. Nelfinavir (Agouron Pharmaceuticals) was the first nonpeptidomimetic compound designed to contain a novel 2-methyl-3-hydroxybenzamide group. Amprenavir (GlaxoSmithKline) is an N,N-disubstituted aminosulfonamide nonpeptide inhibitor with enhanced aqueous solubility compared to previous protease inhibitors and was later replaced on the market with its prodrug, Fosamprenavir, which resulted in lower pill burden. Lopinavir (Abbott Laboratories) is a peptidomimetic protease inhibitor designed for activity against drug-resistant HIV-1 containing mutations at the Val82 residue. Atazanavir (Bristol-Myers Squibb) is an azapeptide protease inhibitor designed to fit the C2 symmetry of the enzyme-binding site and is unique to other PIs as it can only be absorbed in an acidic environment. The resistance profile of atazanavir is also better than previous protease inhibitors. Tipranavir (Boehringer Ingelheim), a nonpeptide inhibitor of protease, was developed from a coumarin template and possesses broad antiviral activity against multiple protease inhibitor-resistant HIV-1. Darunavir (Tibotec, Inc.) is a nonpeptide analog of amprenavir with a critical change at the terminal tetrahydrofuran group, allowing for antiviral activity against amprenavir-resistant HIV-1. Research on new protease inhibitors is directed towards the development of compounds that will not be cross-resistant with other PIs, have a favorable metabolic profile, will not require boosting by RTV, and have a low once-daily pill burden. GlaxoSmithKline discontinued Phase II clinical development of brecanavir due to insurmountable issues regarding formulation. In 2009, GlaxoSmithKline and Concert Pharmaceuticals entered into a collaboration to develop deuterium-containing drugs. CTP518, an analog of atazanavir produced by replacing key hydrogen atoms with deuterium, demonstrated slow hepatic metabolism resulting in an increased half-life and entered Phase I studies in 2010 [79]. CTP518 has the potential to eliminate the need to codose with a boosting agent, such as ritonavir. TM310911, developed by Tibotec Therapeutics, is in Phase II clinical trials with a ritonavir booster. SPI-256, developed by Sequoia Pharmaceuticals in 2008, demonstrated significant potency and a high genetic barrier to resistance *in vitro*. A Phase I study demonstrated safety and tolerability in humans, but SPI-256 development was recently discontinued. SPI-452, a PK enhancer in development by Sequoia Pharmaceuticals, has been shown to increase plasma concentrations of atazanavir and darunavir in Phase I studies without the side effects typically seen with ritonavir as a boosting agent [80]. Cobicistat (GS 9350) by Gilead is a pharmacoenhancer based on CYP3A inhibition, and it represents the PK enhancer in the most advanced development phase. Cobicistat tested against ritonavir with atazanavir plus TDF/FTC and Quad in combination with cobicistat and elvitegravir are all currently in larger Phase III studies. 

Cell-based and biochemical assays are available to evaluate the ability of a compound to inhibit the enzymatic cleavage of viral polyproteins by HIV-1 protease. An HIV-1 protease fluorescence resonance energy transfer (FRET) assay kits are commercially available for biochemical evaluation of potential protease inhibitors [29]. HIV-1 protease activity can be monitored in human cells based on expression of a precursor protein harboring the viral protease fused to the reporter protein GFP [28]. Western analysis of intracellular and virion protein processing can be utilized as well to evaluate HIV-1 protease inhibition.

## 10. Myristoylation Inhibitors 

HIV-1 Gag is synthesized in the cytosol as a precursor protein, p55, and is targeted to the plasma membrane where particle assembly and packaging of viral genomic RNA occur. Modification of p55 at the N-terminal glycine residue with myristic acid, a saturated 14-carbon fatty acid, is essential for targeting p55 to the plasma membrane for HIV-1 assembly. Gag myristoylation consists of two reactions: activation of myristic acid to myristoyl-CoA by acyl-CoA synthetase and transfer of the myristoyl group from myristoyl-CoA to the N-terminal glycine of p55 by N-myristoyltransferase (NMT). Several studies have considered NMT as a potential drug target for the inhibition of HIV-1 assembly. NMT inhibitors have been shown to prevent both membrane binding of Gag as well as virus assembly [81]; however, NMT inhibitors are expected to affect a broad spectrum of cellular processes that depend on protein N-myristoylation for membrane binding. Heteroatom-substituted myristic acid analogs, such as 12-methoxydodecanoic acid, can be used by NMT as alternative substrates for covalent attachment to proteins. The hydrophilic nature of these compounds inhibits membrane binding and function of the modified HIV-1 Gag [82]. The biochemical characterization of these compounds in relation to their effect on HIV-1 remains poorly understood. Dinucleoside fatty acyl prodrugs are being explored for the ability to inhibit HIV-1 replication as a topical microbicide by two mechanisms of action including inhibition of reverse transcriptase and inhibition of the cellular N-myristoyl transferase (NMT) [83].

The levels of myristoylation in cells infected with HIV-1 in the presence and absence of compound can be analyzed by labeling infected cells with [^3^H]myristate and analyzing cell lysates for myristate incorporation into gp41 through immunoprecipitation (IP) with anti-gp41 antibody [81]. Cell-based assays with chronically HIV-1 infected cells can also be used to demonstrate the effects of myristoylation inhibition on proteolytic processing and virus production [84]. 

## 11. Maturation Inhibitors

Maturation inhibitors interfere with the final stage of HIV-1 replication, when viral proteins are assembled, packaged, and released from the host cell membrane to form new virus particles. Bevirimat, a betulinic acid-like compound isolated from the Chinese herb *Syzygium claviflorum*, was purchased by Myriad Genetics from Panacos in 2009, as an inhibitor of HIV-1 maturation. Bevirimat binds to the Gag protein and prevents the critical cleavage of p25 (CA-SP1) between Gag codons 363 and 364 to p24 (CA) and p2 (SP1), resulting in virus particles that lack functional capsid protein and have structural defects rendering them incapable of infecting other cells [85]. Clinical trial data reported in 2009 indicated bevirimat was well tolerated and showed good antiviral activity against HIV-1 with specific Gag protein variations. *In vitro* studies demonstrated the presence of a number of single nucleotide polymorphisms, including H358Y, L363F/M, A364V, and A366T/V, in the CA/SP1 cleavage site that resulted in resistance to bevirimat [86]. Mutations at these sites were not, however, detected in the Phase I and II clinical trials for bevirimat, even in nonresponders. Instead, mutations in the QVT motif of the SP1 peptide (Gag positions 369 to 371) were the primary predictors of failure of response to bevirimat. The comparable potency to other approved HIV-1 drugs, combined with the benefits of oral administration, low probability of drug interactions, and long plasma half-life made bevirimat appear to be a promising new drug candidate. However, Myriad announced in 2010 that it was stopping the development of the maturation inhibitors bevirimat and vivecon.

Maturation inhibitors can be assessed in cell-based assays to evaluate the RNA content and infectivity of virions produced following treatment of infected cells [87]. Electron microscopy can also be used to visualize virions budding from the infected cells following treatment.

## 12. Cellular Targets 

Cells have evolved a number of barriers to resist invading microorganisms. One mechanism that appears to be particularly important in counteracting HIV-1 infection is a group of type 1 interferon-inducible, innate restriction factors that includes tetherin and APOBEC3G. Knowledge of the mechanisms by which restriction factors interfere with HIV-1 replication and how their effects are avoided by HIV-1 in human cells could allow for novel forms of therapeutic intervention. Tetherin is a host protein expressed by many cell types following interferon induction, including CD4-positive T cells, that acts at a late stage of HIV-1 replication to trap mature virions at the plasma membrane by cross-linking to prevent cell-free virus release [88, 89]. Tetherin-retained virions can be reinternalized into the infected cell and targeted to late endosomes where they are destroyed by lysosomal enzymes. However, cell to-cell transmission of HIV-1 is an important mode of dissemination and the possibility of using interferon-based therapy to upregulate the natural antiviral activity of host cells has proven ineffective [90]. APOBEC3G was identified as an inhibitor of HIV-1 replication in cells nonpermissive for replication of HIV-1 mutants lacking a functional Vif gene [91]. APOBEC3G protein can be incorporated into HIV-1 particles through interactions with packaged RNA and the enzyme catalyzes the deamination of deoxycytidines generating minus-strand DNA containing many deoxyuracil nucleotides whose replication results in plus-strand G to A mutations [92]. Hypermutation of HIV-1 DNA can be lethal through deposition of many inactivating missense and nonsense mutations in protein-coding sequences. As previously discussed, RN-18 identified by University of Massachusetts Medical School by high throughput screening of a compound library has been reported to inhibit Vif function by increasing ABOBEC3G concentration within the target cells [93].

Lens epithelial-derived growth factor (LEDGF/p75) is a host protein that binds to HIV-1 integrase and is crucial for viral replication [94]. The mechanism of action is not precisely known but evidence suggests that LEDGF/p75 guides integrase to insert viral DNA into transcriptionally active sites of the host genome. Inhibitors being developed are likely to be highly target specific and less prone to the development of resistance.

Tumor susceptibility gene 101 (TSG101) has been reported to be an essential cellular factor for HIV-1 budding [95]. Inhibiting TSG101 engagement by Gag induces a block of budding virus due to the lipid envelope of nascent particles remaining continuous with the host cell membrane. Mono-clonal antibodies and cyclic peptides have been investigated as inhibitors of TSG101 interactions with Gag.

Second generation nonnucleoside rhodanine derivatives have been reported to have improved inhibition of the human DEAD-box RNA helicase DDX3 leading to anti-HIV-1 activity [96]. DEAD-box proteins have nucleic acid-dependent ATPase activity and are involved in ATP-dependent RNA unwinding. DDX3 has been shown to possess relaxed nucleotide substrate specificity, being able to accept ribo- and deoxynucleoside triphosphates as well as nucleoside analogs. DDX3 incorporates into the nucleocapsids and is an essential cofactor for HIV-1 replication. Studies indicate DDX3 is dispensable for host cell metabolism and would therefore provide an excellent antiviral target with predicted low levels of drug resistance without leading to toxicity from interference of a cellular pathway [97].

Antithrombin III has been reported to activate two host cell interactomes dependent on the NF*κ*B transcription factor, extracellular signal-regulated kinases (ERK), mitogen-activated protein kinase (MAPK), and prostaglandin-synthetase 2 (PTGS2) nodules which have anti-HIV-1 effects [98]. Acceleration Biopharmaceuticals is investigating protein interactomes to identify nodules with host cell factors and pathways for viral inhibition.

Antimicrobial peptides derived from cathelicidins, selected on criteria of length, charge, and lack of Cys since defensins have already been reported to demonstrate anti-HIV-1 effects, are being investigated by the University of Nebraska as potential microbicides [98]. A wide variety of other antimicrobial peptides which have been identified to possess anti-HIV-1 activity are cataloged in the Antimicrobial Peptide Database (APD) maintained by The University of Nebraska [99].

The investigation of host cell factors involved in HIV-1 replication involves profiling of well-characterized signal transduction pathways and antiviral immune responses in HIV-1-infected and uninfected cells treated with test material then building an interactome to identify nodules whose blockage might inhibit viral replication. RT-PCR-based gene arrays are used to determine if cellular gene expression is altered by infected cells treated with a potential inhibitor [14, 100, 101]. Data analysis requires a large data base to define potential nodules responsible for the gene expression alterations. Knockout experiments with siRNA can be used in cell-based assays to confirm the inhibition of HIV-1 replication is due to a particular host cell factor.

## 13. Immunotherapy 

Another approach to treating HIV-1 infection is to strengthen the immune response of infected patients. Immune stimulators are designed to improve overall immune function and include preclinical research on Alferon, human leukocyte-derived interferon alfa-n3 developed by Hemisperx Biopharma, that is currently in Phase III clinical trials [102]. Such approaches like Proleukin, developed by Novartis as recombinant human interleukin-2, have failed in the past to demonstrate stimulation of CD4-positive T cell production in HIV-1-infected patients enrolled in the studies [103]. CYT107, recombinant human interleukin-7 developed by Cytheris, is in Phase II clinical trials with raltegravir and maraviroc with the hope of improving T cell counts in patients classified as immunological nonresponders on antiviral therapy [104]. As a growth factor and cytokine physiologically produced by marrow or thymic stromal cells and other epithelia, IL-7 has a crucial stimulating effect on T lymphocyte development and on homeostatic expansion of peripheral T-cells. Tarix Pharmaceuticals is developing TXA127, angiotensin 1–7, to stimulate bone marrow production of progenitor cells [105]. TXA127 is currently in Phase I studies.

Immunomodulatory compounds are designed to signal immune cells to respond to infection in specific ways and may have direct or indirect antiviral activity. Many immunomodulatory molecules have been shown to reduce cell surface antigen expression using flow cytometry, which resulted in inhibition of virus replication through an entry blocking mechanism. Many immunomodulatory compounds will either inhibit or induce cellular proliferation of specific cell types. In many cases, *in vitro* cell-based assays are not possible, and efficacy will need to be demonstrated using relevant animal models.

## 14. Gene Therapy 

Several gene therapy strategies are being studied in order to construct CD4 cells resistant to HIV-1 infection by a population of anti-HIV-1 antisense RNA producing lymphocytes. Enzo Biochem completed a Phase II clinical trial for HGTV43, a retrovirus vector used to deliver three genes encoding U1/anti-HIV-1 antisense RNA targeting TAR and two separate sites of tat/rev region [106], with results indicating antisense RNA was produced from CD4-positive lymphocytes throughout the 24-month observance but no recent news on the status of HGTV43 could be found. A Phase II clinical trial of VRX496, developed by VIRxSYS, was completed in 2010. VRX496 gene therapy is derived from a lentivirus vector and appears to sustain expression of the delivered genes of interest for a longer period of time compared to previous gene therapies and does not appear to elicit an inflammatory immune response. VIRxSYS is attempting to develop a therapy that will allow HIV-1 patients with undetectable viral loads on HAART to discontinue the antiretroviral treatment and still control their viral load. The VRX496 Phase II study demonstrated a decrease in viral load for 88% of the enrolled HIV-1-infected patients with suppression of HIV-1 viremia for more than 14 weeks in some patients in the absence of HAART [107].

Gene therapies are investigated* in vitro* using cell-based anti-HIV-1 assays that measure reduced virus replication, and effects on specific proteins or RNA can be analyzed by Western or Northern blots [108].

## 15. The Problem of Latent Reservoirs

HIV-1 is known to establish latent reservoirs where the virus is maintained for long periods of time in an essentially quiescent state. Low-rate viral replication also comes from anatomical sites, such as the brain, where drug penetration is limited and only suboptimal drug concentration can be achieved [109]. Studies employing HAART intensification strategies have failed to demonstrate any appreciable reduction in virus load in patients, suggesting the inability to further reduce virus production from these latently infected cells [110]. In recent years considerable interest in the ability to eradicate these latent virus reservoirs and cure HIV-1 infection has evolved. In addition to the HAART intensification studies, efforts have been directed at activating virus production from the latently infected cells to target them for destruction by antiretroviral agents or the host immune system. Compounds developed for this purpose primarily include histone deacetylase (HDAC) inhibitors such as valproic acid, vorinostat, givinostat, and belinostat [111] and nontumor promoting phorbol esters such as prostratin [112]. Compounds which target cellular factors and or regulatory/accessory proteins might also be utilized to target and further reduce virus replication in latently infected cells, such as the transcriptional inhibitors being investigated at ImQuest BioSciences.

The identification and evaluation of compounds which specifically target latently infected cells has primarily utilized the latently infected U1 or ACH-2 cell lines. Primary resting CD4-positive T cells provide the optimal intracellular milieu for establishing latency but are inefficiently infected *in vitro*, since HIV is impaired during reverse transcription and integration. Most primary cell models use one or more rounds of cellular stimulation to remove these blocks, followed by HIV infection during the return to a resting state. Unfortunately, although latently infected nondividing T cells are generated, the process often takes several weeks or months of continuous culture. Investigation into direct infection of resting CD4 T cells by spinoculation has resulted in postintegration latency in these spinoculated cells within 72 h in all CD4 T-cell subsets, including both naive and memory T cells [113]. Cells are sorted by FACS analysis, latent proviruses are activated after additional 72 h of cellular stimulation, and latency can be established and reactivation assessed within 6 days. Using novel reporter viruses, an improved version of this primary CD4 T-cell model has been utilized to study latency in all subsets of CD4 T cells [114]. The ability to target virus in latent reservoirs also requires evaluation in animal models of HIV-1 infection where these reservoirs are established and can be appropriately evaluated. 

## 16. Summary 

Three decades of HIV-1 research have greatly contributed to the knowledge scientists and clinicians have available regarding HIV-1 replication, pathogenesis, and therapeutic strategies. Though great strides have been made in the development of anti-HIV-1 inhibitors targeting various viral enzymes and cellular host factors involved in the virus life cycle, we have learned that multi-drug combinations are necessary for the suppression of viremia and the delayed emergence of drug resistance. More drugs targeting essential virus-specific and/or cellular components of the viral replication pathway and virus transmission are needed to treat and prevent HIV-1 infection. The increasing prevalence of drug-resistant virus strains in patient populations, the increasing incidence of transmission of drug-resistant virus during primary HIV-1 infection, the toxicity of the currently approved therapeutic regimens, and the sometimes difficult regimens that must be followed assure that continued HIV-1 drug development will occur in the foreseeable future. Additionally, development issues must include the ability to safely use drugs in pediatric and pregnant individuals and to specifically target virus in latently infected reservoirs. Finally, increasing emphasis on the eradication of HIV from latent reservoirs in infected individuals will require the development of new and novel treatment strategies. The algorithms available for guiding the screening, identification, characterization, and development of these new compounds have been refined over the years as many thousands of compounds have been evaluated and compounds have been approved for use in humans. Current drug development programs must not only prove the efficacy and safety of the new drug candidates but must also show superiority over existing drugs in the same or similar classes. Thus, drug development must be directed at establishing new and novel drug targets, increasing the potency of existing classes of molecules, decreasing the toxicity or pill burden of existing therapies, or adding new drugs to the HAART regimen with superior combination therapy potential or reduced susceptibility to resistant viruses, including drugs designed specifically to attack existing drug-resistant virus strains. Drug development algorithms in the HIV-1 area must be customizable and highly flexible to assure the ability to characterize novel compounds and therapeutic strategies.

## Figures and Tables

**Table 1:** Anti-HIV-1 screening assays.

Replication event	Assay	Method
Virus Attachment	HeLa-CD4-LT4-*β*-gal cells	HeLa-cell based assay measuring reduction in chemiluminescence of HIV-1-infected cells [16]
	TZM-bl cells	
	gp120/CD4 Ab binding inhibition	Cell based HIV-1 neutralization assay
	gp120 : CD4 ELISA	Biochemical assay with soluble CD4 and monoclonal gp120 antibodies [17]
	gp120/CD4/coreceptor	Cell based, temperature sensitive fusion assay

Fusion and Chemokine Coreceptor Interaction	HL2/3 cells + HeLa-CD4-LTR-*β*-gal cells	Cell based assay measuring reduction in chemiluminesence [16]
	Coreceptor inhibiton	GHOST-cell based assays measuring reduction in virus replication
	Coreceptor typing	PBMC and Macrophage cell-based assays with tropism-specific clinical HIV-1 isolates [18]
	Compound displacement of chemokine ligands	
	Ca^++^flux	

Reverse Transcription	Homopolymer and heteropolymer RT inhibition	Biochemical assay measuring reduction in dGTP-[P32] incorporation [19]
	E/intermediate/late RT products	PCR amplification
	RNaseH inhibition	Biochemical assay [20]
	RT inhibition assays using enzymes with specific mutations	Biochemical dGTP-[P32] incorporation assay [19]

Nuclear localization	2 LTR product in cell nucleus	PCR detection

Integration	Provirus in genomic DNA	PCR detection [21]
	Integrase Complementation	Cell based IN-mutant and Vpr-IN transfection [22]
	Integrase inhibition	Biochemical SPA assay [23]
	Integrase negative virus	

Protein Expression	Northern, Western and flow cytometry	Cell based assays with molecular biology endpoints [24, 25]
	Tat, Rev, and Nef inhibition	Biochemical assays [26, 27]
	Cell-based reporter assay for Rev and Tat function	
	Intracellular p24	CEM-SS cells infected with HIV-1 and lysed to quantitate p24 by ELISA
	LTR-mediated transcriptional activation	

Protease	Intracellular and virion protein processing	Cell based assay with Western analysis [28]
	Polyprotein cleavage	Biochemical FRET assay [29]
